# The added value of text from Dutch general practitioner notes in predictive modeling

**DOI:** 10.1093/jamia/ocad160

**Published:** 2023-08-16

**Authors:** Tom M Seinen, Jan A Kors, Erik M van Mulligen, Egill Fridgeirsson, Peter R Rijnbeek

**Affiliations:** Department of Medical Informatics, Erasmus University Medical Center, Rotterdam, The Netherlands; Department of Medical Informatics, Erasmus University Medical Center, Rotterdam, The Netherlands; Department of Medical Informatics, Erasmus University Medical Center, Rotterdam, The Netherlands; Department of Medical Informatics, Erasmus University Medical Center, Rotterdam, The Netherlands; Department of Medical Informatics, Erasmus University Medical Center, Rotterdam, The Netherlands

**Keywords:** clinical prediction model, electronic health records, machine learning, natural language processing, prognostic prediction

## Abstract

**Objective:**

This work aims to explore the value of Dutch unstructured data, in combination with structured data, for the development of prognostic prediction models in a general practitioner (GP) setting.

**Materials and methods:**

We trained and validated prediction models for 4 common clinical prediction problems using various sparse text representations, common prediction algorithms, and observational GP electronic health record (EHR) data. We trained and validated 84 models internally and externally on data from different EHR systems.

**Results:**

On average, over all the different text representations and prediction algorithms, models only using text data performed better or similar to models using structured data alone in 2 prediction tasks. Additionally, in these 2 tasks, the combination of structured and text data outperformed models using structured or text data alone. No large performance differences were found between the different text representations and prediction algorithms.

**Discussion:**

Our findings indicate that the use of unstructured data alone can result in well-performing prediction models for some clinical prediction problems. Furthermore, the performance improvement achieved by combining structured and text data highlights the added value. Additionally, we demonstrate the significance of clinical natural language processing research in languages other than English and the possibility of validating text-based prediction models across various EHR systems.

**Conclusion:**

Our study highlights the potential benefits of incorporating unstructured data in clinical prediction models in a GP setting. Although the added value of unstructured data may vary depending on the specific prediction task, our findings suggest that it has the potential to enhance patient care.

## INTRODUCTION

Electronic health record (EHR) databases form a rich data source for building prognostic clinical prediction models, and their development and validation have become increasingly important in clinical research.^1–3^ A prognostic model predicts which patients, among a target population, using predictors measured during a prior observation time window, will experience a clinical outcome during a window of time in the future. While model development typically focuses on the use of structured EHR data, such as coded conditions, prescriptions, and measurements, most detailed and extensive clinical information is commonly stored in the vast number of clinical notes from physicians, nurses, or other caregivers, used for documentation or communication: the unstructured or free-text data.^4^^,^^5^ Transforming extensive volumes of clinical text into structured numerical features for model development is a resource-intensive process, making the use of text data in prediction models complex in comparison to structured data. However, incorporating detailed information from clinical narratives has the potential to improve the predictive performance of the model, as it provides a more complete and accurate picture of the patient’s health.

Our previous research reviewed the use of text data in prognostic prediction models in recently published literature and found that, in addition to structured data, text data improved the performance of most models.^6^ However, there are still several knowledge gaps that can be addressed. First of all, assessing generalizability remains important in model development,^7^^,^^8^ but the majority of developed and published prediction models is not externally validated.^2^^,^^6^ While external validation of structured data models is increasingly feasible, for example, by the use of common standardized clinical concepts and the Observational Medical Outcomes Partnership Common Data Model (OMOP CDM),^3^ external validation of models using text data is still complicated. This is due to the use of different languages or clinical sublanguages, the lack of a common structured format, and noisy and subjective text containing abbreviations and typos. Furthermore, the focus of clinical prognostic prediction and natural language processing (NLP) research has been on English and hospital-care data.^6^^,^^9^ Only a few studies have recently incorporated Dutch clinical text in the development of prognostic prediction models, predicting life expectancy,^10^ risk of preterm birth,^11^ and the risk of inpatient violence.^12–15^ All these studies found the use of text data beneficial, yet were also primarily conducted in a hospital-care setting. However, other studies have utilized Dutch general practitioner (GP) notes to predict the risk of lung cancer^16^ and falls in elderly patients,^17^ but with no comparison to models using structured data. Previous NLP research has demonstrated the accurate extraction of important information from Dutch patient notes^18–20^ and the development of large clinical language models,^21^^,^^22^ and highlighted the potential added value of text data in prognostic prediction.

Primary care in the Netherlands serves as the first point of contact for patients seeking medical attention.^23^ As such, GPs play a crucial role in detecting and addressing health concerns early to prevent disease progression and improve patient outcomes, often for patients with multiple or chronic conditions.^24^ Additionally, GP settings generally have limited resources, which need to be used efficiently to maximize patient benefits. Prediction models can aid the GP to prioritize care and effectively allocate resources. To realize these potential benefits of prediction models, more large-scale research on model development and validation in the primary care setting is needed.^25^ However, GP patient data in the Netherlands are managed and stored locally or by third-party EHR system vendors, not centrally. Therefore, nationwide observational research is challenging due to diverse data models and the privacy-sensitive nature of the text data. Furthermore, the clinical GP sublanguage used in the Dutch notes makes understanding the text data difficult, with the notes often containing abbreviations, spelling errors, and short forms. In contrast, the notes in the Medical Information Mart for Intensive Care (MIMIC) III hospital dataset^26^ commonly used for English clinical NLP research are more straightforward to interpret and assess. Therefore, developing prediction models using Dutch GP notes with this distinct sublanguage brings an additional challenge.^27^

This work aims to explore the value of Dutch EHR text data, in combination with structured data, for the development of prognostic prediction models in a GP setting. We create prediction models for 4 common clinical problems using observational data from different EHR systems in a large Dutch GP EHR database, sparse text representations, and common prediction algorithms. We evaluate the models using internal and external validation to assess their generalizability. Additionally, we investigate the effect of different text representations and prediction algorithms on prediction performance.

## MATERIALS AND METHODS

### Dataset and setting

The Integrated Primary Care Information (IPCI) database^28^ is a database containing longitudinal data from EHRs of around 350 GP practices, using 6 different EHR systems, throughout the Netherlands. The database contains 2.5 million patient records from 1992 to 2022 with a median patient follow-up duration of 4.8 years. The 1.4 million active patients comprise 8.1% of the Dutch population. The database has been converted to the OMOP CDM, enabling collaborative research in a large international network of databases using standardized analytics.^29^ This research was approved by the IPCI governance board and registered under code 2023-03.

### Prediction problems

We focused on 4 different prediction tasks, for which we formulated a prediction problem with the aid of 2 clinical experts, according to a standardized patient-level prediction framework^3^ and defined it using OMOP CDM cohort definitions, promoting transparency and reproducibility. Table 1 summarizes the target and outcome event, the time-at-risk, and the observation period for each of the following problems.

**Table 1.** Overview of the prediction problems in the 4 prediction tasks.

	Hospital readmission	End-of-life care	Asthma exacerbations	Mortality in COPD
Target event	Adult (18+) patients with a hospital discharge between January 2016 and January 2021.	Older (60+) patients with a first visit between January 2016 and January 2021 and a prior diagnosis of heart failure, chronic obstructive pulmonary disease, or cancer.	Adult (18+) patients with a new asthma diagnosis under medication between January 2015 and January 2020.	Adult (18+) patients with a new diagnosis of COPD between January 2015 and January 2020.
Outcome event	Hospital readmission	First end-of-life conversation	Asthma exacerbation	All-cause mortality
Time-at-risk	2-30 days (1 month)	1-365 days (1 year)	1-730 days (2 years)	1-730 days (2 years)
Observation period	365 days prior to discharge	365 days prior to the visit	365 days prior to the medication start	365 days prior to the diagnosis

Abbreviation: COPD, chronic obstructive pulmonary disease.

- **Hospital readmission—**A common problem in hospital-centric prediction research.^2^^,^^6^^,^^21^^,^^22^ For every adult patient discharged from the hospital, we predicted the risk of another hospital admission between 2 and 30 days after discharge, starting on day 2 to exclude in-hospital transfers.
- **End-of-life conversation—**A problem that is specifically of interest to GPs who aim to identify patients with complex palliative needs.^23–25^ At every first yearly GP visit of patients older than 60 years, with a history of heart failure, chronic obstructive pulmonary disease, or cancer, we predicted the risk of the first end-of-life conversation within 365 days after the visit.
- **Asthma exacerbations—**Asthma exacerbations are associated with poor quality of life and a prediction model could help identify patients for more frequent monitoring or step-up of treatment.^30–32^ We estimated for newly diagnosed adult asthma patients who receive treatment the risk for asthma exacerbations within 2 years of diagnosis.
- **Mortality in chronic obstructive pulmonary disease (COPD) patients—**COPD is a leading cause of death and decreases the quality of life in advanced stages. Palliative care is suggested in the final year of life, but uptake is low because death is difficult to predict.^29^ We predicted for newly diagnosed COPD patients the mortality risk within 2 years of diagnosis.

For all 4 problems, an observation period of 365 days before the target event was used for covariate extraction. The study period of each prediction problem, the time in which a patient could enter the study, was defined by the last 5 years in the database before the required time-at-risk. Furthermore, patients with less than 365 days of observation time and patients without follow-up, that is, patients who died or left the practice before the end of the time-at-risk, were excluded. Only in the hospital readmission task, a patient could have multiple observations—hospital admissions—in the dataset, in the other tasks each included patient had 1 observation. Full cohort definitions, including standardized codes, can be found in the supplementary material.

### Feature extraction

**Figure 1.**
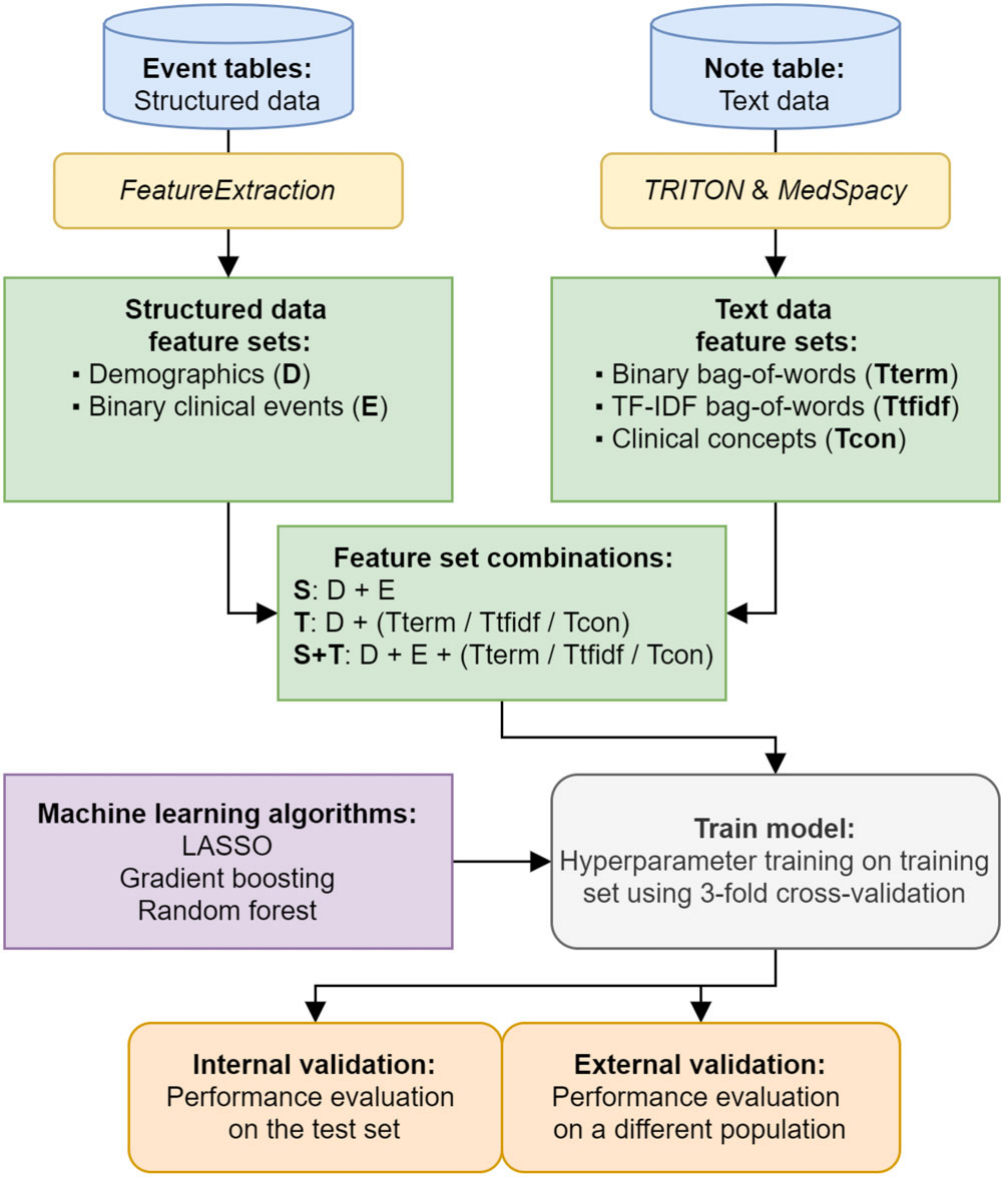
Overview of the experimental setup, showing the feature extraction workflow from both structured data and text data, feature set combinations, model development, and evaluation.

Features were extracted during the observation period of each prediction task, from both the structured data and the unstructured data, in a window of 365 days and 30 days before the target event. An overview of the feature extraction workflow, including model development and evaluation is shown in Figure 1.

#### Structured data

Feature sets from structured data were generated using the *FeatureExtraction* R-package (https://ohdsi.github.io/FeatureExtraction/), a tool for generating features for a cohort in the OMOP CDM. These features included patient demographics—age and sex—and occurrences of all clinical events: conditions, drugs, measurements, and procedures. This resulted in sparse binary feature vectors indicating the occurrence or not of each medical event in the observation period. We created 2 structured data feature sets, 1 with only demographic features (D) and 1 with all clinical event features (E).

#### Unstructured data

All notes within the 365-day observation period were used and all types of clinical notes were considered for analysis, including GP notes and communication between care providers, such as radiology reports and hospital and emergency department discharge letters. Identifiable information was removed from the text or pseudonymized at the data source.^28^

#### TRITON

**Figure 2.**
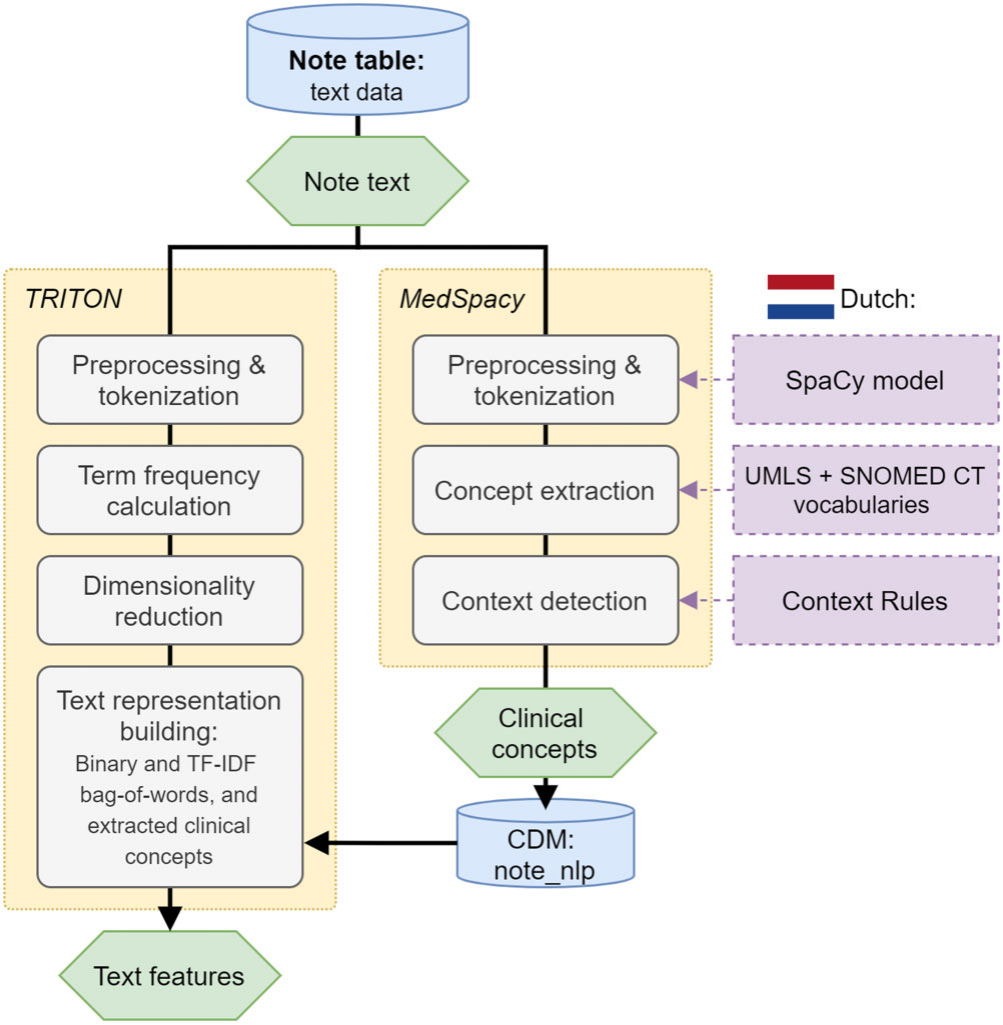
Visualization of the *TRITON* and *MedSpacy* pipelines to process raw text data from the note table in the OMOP CDM and generate text-based features for prediction models.

We developed the Text Represented In Terms Of Numeric-features (*TRITON*) R-package (https://github.com/mi-erasmusmc/Triton), a *FeatureExtraction* extension, to construct text-based numeric feature vectors from free-text notes in the OMOP CDM *note* table. *TRITON* provides a customizable, modular NLP pipeline for text preprocessing, tokenization, and vectorization (see Figure 2). The pipeline settings are saved with the processed results for sharing and reproducibility. In this work, we used 2 different text representations: individual words (or bag-of-words) and clinical concepts.

#### Bag-of-words

To create bag-of-words features, we first combined all the text from the individual notes into a single document for each observation period. We preprocessed the text by replacing numeric and new line characters and underscores with empty spaces. The text was tokenized into individual terms using a simple tokenizer, splitting the text at spaces and punctuation. We calculated the term and document frequencies and reduced the dimensionality of the resulting matrix by removing noninformative tokens appearing in more than 80% or less than 0.1% of the documents. For each observation, we created 2 vectors: one that represented the binary occurrence of each term (Tterm) and another that represented the term frequency-inverse document frequency (TF-IDF) (Ttfidf).

#### Clinical concept extraction

To extract clinical concepts from the text, we used *MedSpacy*,^33^ a clinical text processing toolkit that enables Unified Medical Language System (UMLS) concept extraction and context detection (Figure 2). *MedSpacy* has been designed to process text in English, but we replaced the English dependencies with Dutch resources. As a vocabulary for the clinical concept extraction, we used the Dutch translation of the Systematized Nomenclature of Medicine Clinical Terms (SNOMED CT) (https://www.snomed.org/member/netherlands), maintained by NICTIZ, the Dutch National IT Institute for Healthcare, combined with 5 other Dutch vocabularies from the UMLS (see Table S1). Furthermore, we used existing Dutch context rules to detect whether a concept was negated, was mentioned in a hypothetical or historical context, or concerned the patient or someone else.^34^^,^^35^ The Dutch spaCy model (https://spacy.io/models/nl) was used for sentence splitting and tokenization. This combination of resources enabled the extraction of concepts and their context from Dutch clinical text. Using *TRITON*, we converted the extracted concepts for each observation into a binary feature vector that indicated their presence during the observation period (Tcon), in a similar fashion as for the structured data features.

### Machine learning algorithms

We focused on 3 machine learning algorithms that are commonly used in predictive modeling^2^^,^^6^^,^^36^^,^^37^: L1 regularized logistic regression (LR) or Lasso, extreme gradient boosting (XGB), and random forests (RF). An overview of the algorithms’ hyperparameters and their value range is presented in Table S2. Hyperparameters were optimized using 3-fold cross-validation. Data-driven model training and evaluation were performed using the *PatientLevelPrediction* R-package,^3^ the tool for building and validating patient-level predictive models using OMOP CDM data. Data-driven implies that the information extraction process from the text or structured data was unrestricted and not limited to anticipated significant features.

### Internal and external validation

**Figure 3.**
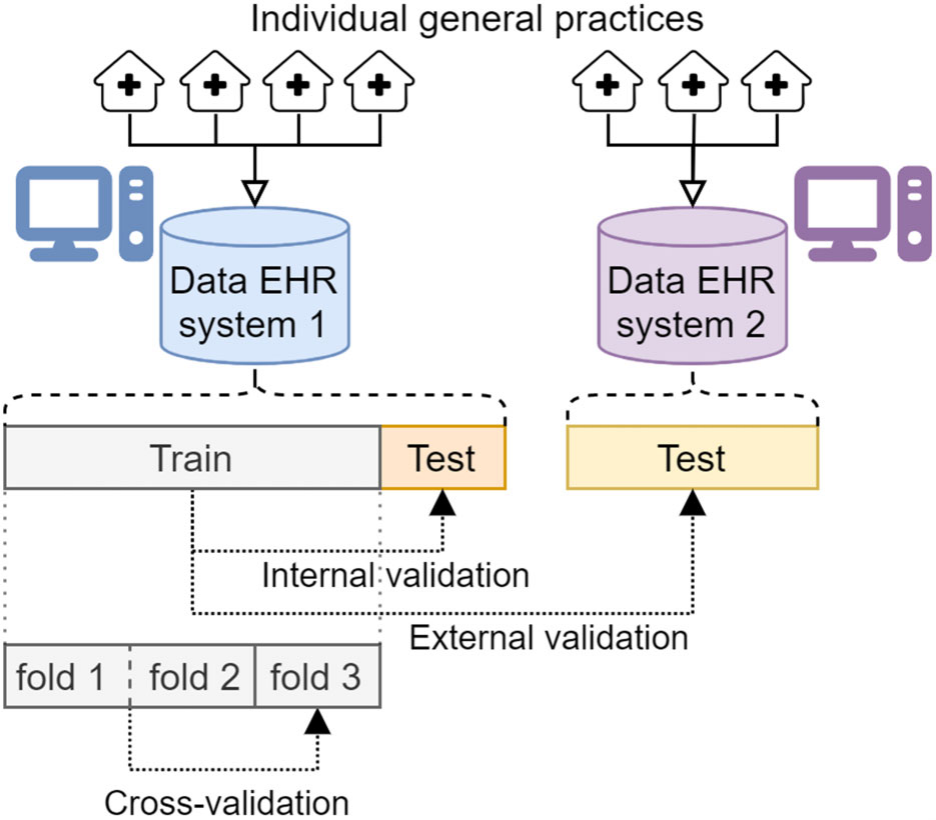
Overview of internal validation, external validation, and cross-validation within and between groups of individual general practices that use two different EHR systems.

We performed both internal and external validation (Figure 3): internal validation on data from practices that use the same EHR system, and external validation on data from practices that use a different EHR system. Specifically, for each prediction task, we ranked the 6 EHR systems according to the number of observations for that task and only used the data from the 2 EHR systems with the highest number of observations. We split the observations in the EHR system with most observations into a 25% test set and a 75% training set on a subject level. Every model was trained on the training set and then internally validated on the test set. For external validation, we evaluated the resulting models on all the observations in the second EHR system dataset, assessing their generalizability over a different population.

### Feature set combinations and experimental setup

We conducted several experiments to evaluate the contribution of text data to prediction models by training them with different combinations of structured and unstructured data feature sets (Figure 1). Specifically, we used the demographic features (D), the clinical event features (E), and 3 text representation feature sets: binary bag-of-words (Tterm), TFIDF bag-of-words (Ttfidf), and extracted clinical concepts (Tcon). We combined these features to create 7 feature set combinations: a structured data feature set (S: D+E), 3 text-data feature sets (T: D+Tterm, D+Ttfidf, and D+Tcon), and 3 combined-data feature sets (S+T: D+E+Tterm, D+E+Ttfidf, and D+E+Tcon). Demographic features were considered separate from clinical events and combined with text representations in the textual data feature set because they are always available for every patient. In total, we trained and evaluated 21 models per prediction task, using 7 feature set combinations and 3 prediction algorithms, 84 models in total. The different feature set combinations and methods are also summarized in Table S3.

### Model evaluation

We used 2 metrics, the area under the receiver operating characteristic curve (AUROC) and the area under the precision-recall curve (AUPRC), to evaluate and compare our models’ performance in terms of class discrimination. Additionally, we calculated the precision, recall, and F1-score of each model for the probability threshold that maximized the F1-score, and we measured model calibration using the Brier score. To determine whether the performance medians for structured (S), text (T), and combined (S+T) feature sets were significantly different across different text presentations and machine learning algorithms, we conducted 2-sided Bonferroni-adjusted Wilcoxon tests. Furthermore, we evaluated the overall effect of the text presentations and machine learning algorithms on the model performance by grouping the models by prediction task and feature set combination, calculating the median of evaluation metric values for each group, and subtracting this median from the values in that group, also called median-centering.

In addition to evaluating the performance of models using the global evaluation metrics above, we also sought to measure the degree of predictive multiplicity between models. Predictive multiplicity refers to differences in predictions that are made for a given outcome, based on different models or variables.^38^^,^^39^ Even if models have a similar performance as measured by AUROC or AUPRC, their predictions may still differ. This is because a similar performance value can be obtained by correctly identifying different sets of observations. One way to assess predictive multiplicity is to calculate the correlation between the predictions made by 2 models. A strong correlation indicates a low degree of predictive multiplicity and vice versa. Additionally, besides the predictive value, explainability of the models is important to gain trust in the predictions and to identify which information in text data is used to make these predictions.^40^ The global explainability of the models was assessed by identifying the most important features. The importance of features for regularized LR was based on their beta values, while the mean decrease in impurity was used for random forest, and the average gain for gradient boosting.

## RESULTS

### Population characteristics

The population characteristics and the characteristics per observation for each prediction task and EHR system are presented in Table 2. The 2 largest EHR systems for the end-of-life conversations, asthma exacerbation, and COPD mortality tasks were the same, EHR systems A and B. In the readmission task, EHR systems C and D had the most observations. The outcome-to-observation ratio differed per prediction task but was similar between EHR systems. No large differences in age and sex were found. Some differences were seen for the median number of clinical events recorded during the observation periods. For instance, EHR system A showed a higher median number of conditions than EHR system B, but a lower median number of drugs. EHR systems C and D, used for the hospital readmission task, exhibited a higher median number of extracted concepts as well as words and sentences, mainly due to an increased number of communication notes between GP and hospital. However, the number of clinical events was comparable with the other tasks.

**Table 2.** Cohort characteristics and observation per prediction task and EHR dataset. Each observation consisted of patient notes from the observation period of 365 days.

	Asthma exacerbations		Mortality in COPD		End-of-life care		Hospital readmission	
	EHR 1 (A)	EHR 2 (B)	EHR 1 (A)	EHR 2 (B)	EHR 1 (A)	EHR 2 (B)	EHR 1 (C)	EHR 2 (D)
Cohort characteristics								
Number of GPs	114	99	114	96	111	114	73	43
Number of patients	12 385	5086	2067	1749	34 359	15 470	37 443	27 897
Number of observations	12 385	5086	2067	1749	34 359	15 470	55 152	38 633
Number of outcomes	4827	2362	194	158	1490	704	5736	3997
Outcome-to-observation ratio	0.39	0.46	0.09	0.09	0.04	0.05	0.10	0.10
Average age	46.8	46.7	65.5	65.7	73.2	73.7	63.2	62.0
Sex, percentage of male	61	64	48	48	51	50	52	53
Characteristic per observation								
Median no. of (distinct) conditions	13 (6)	7 (5)	21 (9)	9 (6)	20 (8)	8 (6)	7 (4)	8 (4)
Median no. of (distinct) drugs	6 (5)	8 (7)	7 (5)	9 (7)	7 (5)	10 (7)	8 (7)	9 (8)
Median no. of (distinct) measurements	9 (7)	15 (12)	25 (20)	24 (20)	25 (19)	27 (21)	23 (16)	20 (14)
Median no. of (distinct) procedures	0 (0)	2 (1)	0 (0)	3 (1)	1 (1)	3 (2)	1 (1)	2 (1)
Median no. of (distinct) extracted concepts	83 (51)	101 (60)	96 (57)	109 (64)	113 (65)	134 (76)	623 (221)	574 (210)
Median no. of notes	28	36	39	44	39	47	47	56
Median no. of characters	2147	2605	2882	3222	2907	3546	16079	14757
Median no. of (distinct) words	318 (211)	389 (244)	430 (266)	480 (282)	430 (244)	530 (302)	2207 (786)	2058 (774)
Median word length in characters	4	4	4	4	4	4	5	5
Median no. of sentences	59	77	82	93	80	101	278	263
Median sentence length in words	5	5	5	5	5	5	8	8
Median no. GP notes	25	34	34	41	34	44	36	42
Median no. communication notes	3	2	5	2	6	2	10	13

Abbreviations: EHR, electronic health record; GP, general practitioner.

### Comparing feature combinations

**Figure 4.**
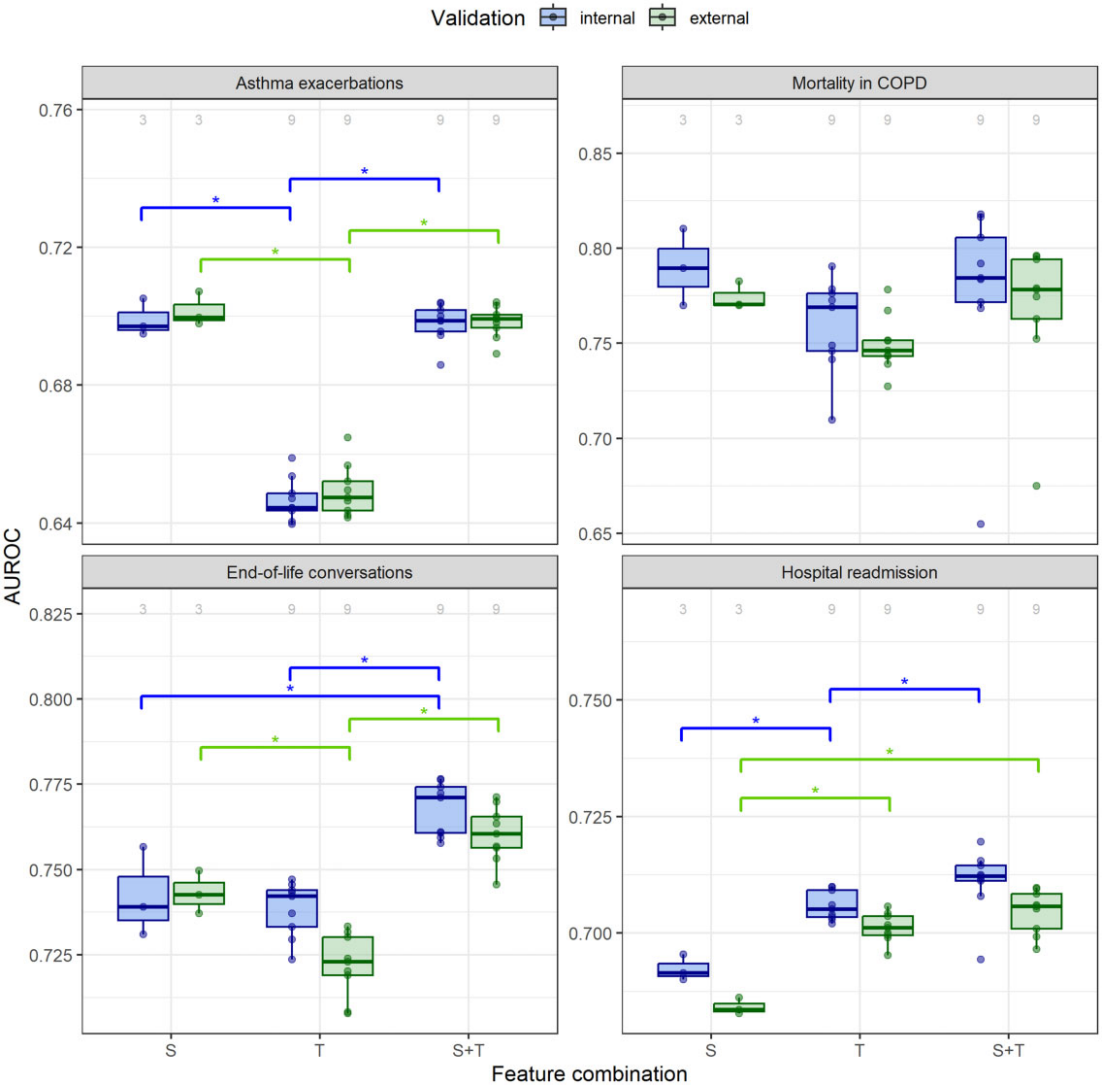
Distribution of the AUROC values of the internal validation (blue) and external validation (green) for models trained using the different feature combinations: structured feature set (S), text feature sets (T), and combined feature sets (S+T), per prediction task. The number of models in each boxplot is indicated above the boxplot. The significant Bonferroni-adjusted Wilcoxon test results between the feature combinations are shown above the boxplots, where “*” indicates a *P* value <.05. The points represent the underlying data.

All 84 trained models were both internally and externally validated. Figure 4 visualizes the internal and external validation performances measured by the AUROC value for each prediction task and feature combination, over the different text representations and machine learning algorithms. Figure S1 and Figure S2 depict the same for the AUPRC and Brier evaluation metrics, respectively. The complete evaluation results for both internal and external evaluation of all models are available in the supplementary material.

The structured data models had acceptable predictive performance in all tasks, with AUROC values ranging from 0.68 to 0.79. The relative predictive performance of text data models compared to structured data models varied depending on the task. In the hospital readmission task, text data models performed better than structured data models. In the asthma exacerbation, end-of-life conversation, and mortality in COPD prediction tasks, they performed similarly or worse. In situations where text data models performed worse than structured data models, the combined data models showed no significant difference in performance as compared to structured data models. However, in the hospital readmission and end-of-life conversation prediction tasks, where text data models outperformed or performed similarly to the structured data models, the combined data model displayed a higher median performance compared to both the structured and text data models in the external validation or the internal validation. Similar results were observed for the AUPRC evaluation metric (Figure S1). Overall, external validation performance was mostly comparable or lower than internal validation. The Brier scores (Figure S2) showed that in the hospital readmission prediction task, text data models were better calibrated than structured data models.

### Comparing text representations and machine learning algorithms

**Figure 5.**
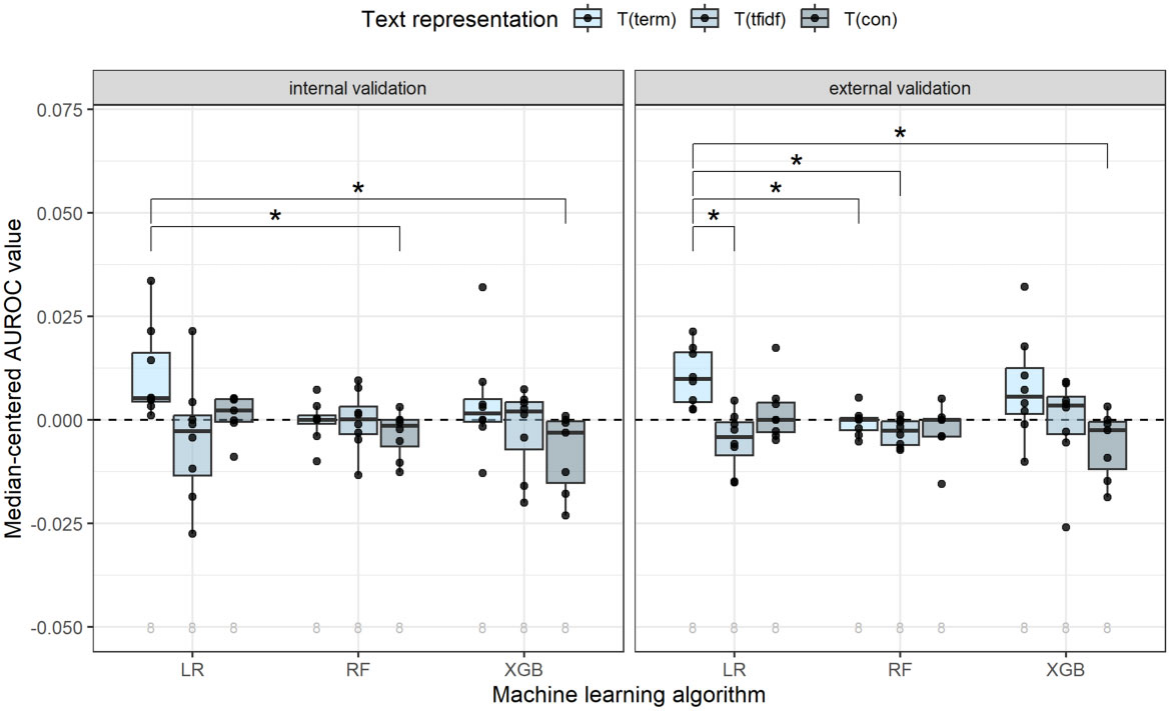
Distributions of the median-centered AUROC value for the different text representations and machine learning algorithms. The plots on the left-hand side present the internal validation and the plots on the right-hand side present the external validation. The number of models is noted below each boxplot. The significant Bonferroni-adjusted Wilcoxon test results between the text representation and machine learning algorithm combinations are shown above the boxplots, where “*” indicates a *P* value <.05.

The performance difference between different combinations of text representations and machine learning algorithms, for both internal and external validation and measured by the AUROC, is visualized in Figure 5. Figure S3 depicts this performance comparison for the AUPRC. Two-sided Bonferroni-adjusted Wilcoxon tests across all combinations showed that there is a significant external validation AUROC improvement of regularized LR with binary bag-of-words features over regularized LR and RF with TF-IDF bag-of-words features and XGB with clinical concept features. Other effects that were significant in the internal validation were lost in external validation. No significant differences were found for the AUPRC metric.

### Predictive multiplicity between models

**Figure 6.**
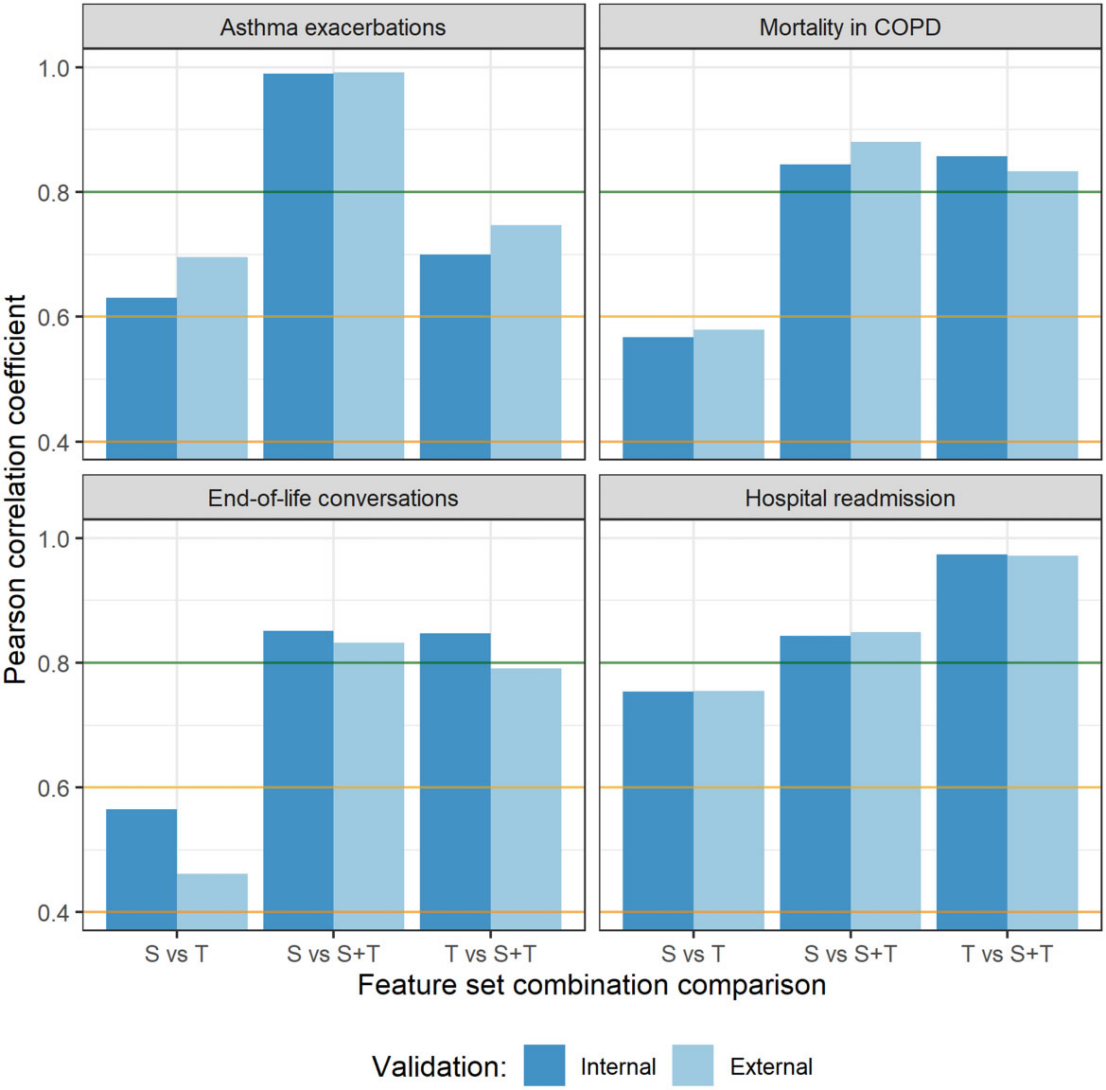
Pearson correlation coefficients between predicted probabilities of the structured data (S), text data (T), and combined data (S+T) models for each prediction task in the internal and external validation. Horizontal lines indicate thresholds for, from top to bottom, a very strong correlation (*r* > .8), strong correlation (.8 > *r* > .6), and moderate correlation (.6 > *r* > .4).

To answer the question of whether the structured data, text data, and combined data models predict the same outcomes for the same patients, we measured the predictive multiplicity by calculating the correlation between the models’ predicted probabilities. Figure 6 presents the Pearson correlation coefficients between predicted probabilities of structured, text, and combined data models, for each prediction task in the internal and external validation, averaged across text representations and prediction algorithms. Correlations between text data and structured data models were found to be moderate to strong in most tasks; in the end-of-life conversation task the correlation was lowest. This indicates that some multiplicity existed between the text data models and structured data models. In the mortality in COPD and end-of-life conversation prediction tasks, the combined data models were equally highly correlated to the text and structured data models. In the asthma exacerbation task, the combined model had a higher correlation to the structured data than to the text data model and the opposite was observed in the hospital readmission task. No large differences were observed between the internal and external validation.

### Feature importance

The supplementary material contains the top 10 features with the highest absolute importance in each model. As an example, Table 3 displays for each prediction task the top 5 features of the text-based regularized LR models using binary bag-of-words features, a method combination that performed relatively well (Figure 5). The association between most terms and the outcome is expected, such as between “lung carcinoma” or “metastasized” and future end-of-life conversations, but some terms pose interpretation challenges. Furthermore, it should be noted that the features only indicate an association with the outcome in the database, but not necessarily a causal relationship.

**Table 3.** Lists of the 5 features with the largest absolute beta values in the text-based regularized logistic regression models using binary bag-of-words features. English translations are provided where necessary. Note that these features only reflect an association with the outcome in the database, not a causal relationship.

Asthma exacerbations		Mortality in COPD		End-of-life care		Hospital readmission	
Term	Beta	Term	Beta	Term	Beta	Term	Beta
*prednison*	.412	*visite* (visit)	.762	*longcarcinoom* (lung carcinoma)	.662	*ct* (computed tomography)	.212
*eczeem* (eczema)	.359	*keer* (times/turn)	−.633	*gemetastaseerd* (metastasized)	.623	*oncologie* (oncology)	.194
*prednisolon*	.287	*cordis*	.591	*palliatieve* (palliative)	.467	*tumor*	.162
*oor* (ear)	.262	*onderzoek* (examination)	.588	*meta* (metastasized)	.425	*specialisme* (specialism)	.154
*kenacort*	.192	*astma* (asthma)	−.460	*copd*	−.359	*vaatchirurg* (vascular surgeon)	.153

Abbreviation: COPD, chronic obstructive pulmonary disease.

## DISCUSSION

### Value of unstructured text

In this study, we explored the added value of Dutch unstructured text data for the development of prognostic prediction models in a GP setting. We created and evaluated prediction models for 4 prediction tasks on common clinical problems using both structured and unstructured data features extracted from Dutch observational GP data. For each prediction task, we compared the difference in average performance between models trained on text data and models trained on structured data. One task showed a higher performance for text data models and 3 tasks showed a similar or lower performance. Nonetheless, the ability to train well-performing models for some of the tasks using only unstructured text highlights the valuable information extracted from clinical narratives. This result was also found by other recent studies predicting future events using only GP notes.^16^^,^^17^ On average, combined data models performed better than models using only structured data or text data in 2 out of 4 prediction tasks. This suggests that here the combination of features leverages the strengths of both data types to make more accurate predictions. Although not all differences observed were statistically significant, there is a discernible pattern indicating that the addition of information from text to structured data enhances predictive performance. These findings align with previous studies that compared the use of text and structured data in predictive models in other languages and settings.^6^

### Methods comparison

Comparison of performances of different prediction algorithms and text representation combinations indicated that models using LR and binary bag-of-words features performed significantly better than 3 out of the 8 other combinations. This shows that using a relatively simple machine learning algorithm and a sparse text representation prediction models can be built that outperform more complex bagging or boosting tree-based algorithms with more dense text features, which was also found by others.^37^^,^^41^ However, differences were small and other method combinations showed similar performances.

### Data type information difference

We found some predictive multiplicity between the structured and unstructured data models in most tasks, suggesting that the text data offered information not captured by structured data and the other way around. This could explain why combining information from both structured and text data improves predictive performance in some prediction tasks. However, the performance of combined data models did not show improvement in all prediction tasks where we found a relatively high predictive multiplicity. Therefore, factors influencing the performance of a model that combines text and structured data, remain to be explored. Potential factors include the quantity and quality of information in both data types. For instance, while longer clinical notes and more coded conditions potentially contain more relevant information, poorly written text or wrongly coded events would reduce the predictive value.

### Strengths and limitations

We used in this study a limited number of sparse text representations and common prediction algorithms. While more advanced NLP and machine learning methods, such as word embeddings and deep learning, could potentially improve the accuracy of our predictions, we intentionally limited the number of studied methods to keep the number of models reasonable, given our consideration of multiple prediction tasks and external validation. Moreover, our main goal was to demonstrate the added value of text data and even with these limited methods, we were able to show an improvement in predictive performance in certain cases. The strength of our study lies in the fact that we aimed to assess the added value of the unstructured text in 4 diverse prediction tasks, with different outcome time-at-risk periods and numbers of observations, which were not selectively chosen to perform well with unstructured data. Furthermore, we chose to report the performance of the models over multiple method combinations, instead of only reporting and comparing the best model within each group, providing a more complete picture. As model explainability and trustworthiness are crucial in clinical prediction models, we evaluated their global explanations by examining their feature importance.^40^ We found that, generally, the associations between the features and the outcome can be expected on a clinical basis; however, some associations require a more thorough inspection. This demonstrates that the feature importance values provide valuable insights into the information employed by the models, which can aid in understanding or refining the model. Nonetheless, additional post-hoc explanation techniques, such as local interpretable model-agnostic explanations^42^ and Shapley values,^43^ could also be utilized to further enhance confidence in the model predictions.

The study was conducted on data in a non-English language, Dutch, and on a GP database, contributing to previous prediction research that was mainly focused on databases in a hospital setting and the English language. The transformation of independent datasets from different GP EHR systems into the OMOP CDM enabled our large-scale observational research and the external validation of text-based models across GPs in the Netherlands. Additionally, we used standardized feature extraction and prediction modeling tools, promoting reproducibility and transparency. Finally, it is important to note that the overall performance of the models may not be sufficient for clinical application yet, and further improvement, using alternative methods or more data, is certainly needed. However, with continued refinement and evaluation of their clinical utility, these models hold promise as valuable screening tools, decision support aids for GPs, and for managing population health.

### Future work

The performance of models using unstructured text data varied across the 4 prediction tasks. Further research is needed to explore reasons for these differences, to understand the information differences and overlap between structured and text data, and to develop different strategies for combining these data types. For example, an ensemble model strategy could be considered, where predictions of the separate models are aggregated. Additionally, we did not use deep learning or dense text representations, as these models can be prone to overfitting, in particular on a few thousand observations.^44^ However, deep learning and dense text representation methods may find more complex and contextual relations in both unstructured text and structured data and should be a focus of future research. Furthermore, it is crucial to promote clinical NLP research in languages other than English and to further explore methods for the external validation of text-based models across databases in multiple languages. Possible methods include normalizing information between the languages through translation, training multilingual models, or extracting clinical concepts using a multilingual clinical ontology. Taking into account language-specific cultural and linguistic factors across populations and databases may lead to more robust models and more relevant results.^9^

## CONCLUSION

In conclusion, our study demonstrated the feasibility and potential value of incorporating unstructured text data from a large GP database, originating from multiple EHR systems, in clinical prognostic prediction. The text data models showed higher or similar performance as compared to models based solely on structured data in 2 prediction tasks for which further combining structured and text data resulted in improved performance. This shows that the added value of incorporating unstructured text data in clinical prediction models may vary depending on the specific prediction problem. No large performance differences were found between different text representations and prediction algorithms, but a simple LR model with a binary bag-of-words input outperformed several more complex models. Furthermore, the predictive multiplicity found between models trained on structured and text data suggests that the information in these data types differs. Therefore, it is essential to explore factors influencing the performance of combining these data types or alternative methods of combining the individual models. Our study also demonstrated the value of clinical NLP research in a language other than English, the feasibility of externally validating text-based prediction models across EHR systems, and assessing the model explainability using the global feature importance.

## Supplementary Material

ocad160_Supplementary_Data

## Data Availability

The aggregated data used for generating the results, conclusions, and figures/tables in this study are available as supplementary data. The OMOP-CDM format of the IPCI database enables collaboration and facilitates participation in large-scale studies, requiring cooperation with the Department of Medical Informatics at Erasmus University Medical Center and approval from the IPCI governance board.
