# Supplementary material for: The added value of text from Dutch general practitioner notes in predictive modeling
Source: J Am Med Inform Assoc. 2023 Aug 16;30(12):1973–84. doi: 10.1093/jamia/ocad160 (PMC10654855; doi:10.1093/jamia/ocad160)
Supplement: ocad160_Supplementary_Data [file ocad160_supplementary_data.zip › ocad160_Supplementary_Data/PlpUnstructuredData_supplement_CohortDefinitions.docx]

## Hospital Readmission: target

**Cohort Entry Events**

**People may enter the cohort when observing any of the following:**

- visit occurrences of 'inpatient visit'.

**Inclusion Criteria**

- Age >= 18
  - Entry events with the following event criteria: who are >= 18 years old.
- Time period
  - Entry events with the following event criteria: starting on or after January 1, 2016 and ending before January 1, 2021.

**Cohort Exit**

- The cohort end date will be offset from index event's end date plus 0 days.

**Cohort Eras**

- Entry events will be combined into cohort eras if they are within 0 days of each other.

**Concept Set Definitions**

- inpatient visit

| Concept Id | Concept name | Domain | Vocabulary | Excluded | Descendants | Mapped |
| --- | --- | --- | --- | --- | --- | --- |
| 9201 | Inpatient Visit | Visit | Visit | NO | NO | NO |

OHDSI vocabulary version: v5.0 04-FEB-22

## Hospital Readmission: outcome

**Cohort Entry Events**

**People may enter the cohort when observing any of the following:**

- visit occurrences of 'inpatient visit'.

**Inclusion Criteria**

- Age >= 18
  - Entry events with the following event criteria: who are >= 18 years old.
- Time period
  - Entry events with the following event criteria: starting on or after January 1, 2016.

**Cohort Exit**

- The cohort end date will be offset from index event's start date plus 0 days.

**Cohort Eras**

- Entry events will be combined into cohort eras if they are within 0 days of each other.

**Concept Set Definitions**

- inpatient visit

| Concept Id | Concept name | Domain | Vocabulary | Excluded | Descendants | Mapped |
| --- | --- | --- | --- | --- | --- | --- |
| 9201 | Inpatient Visit | Visit | Visit | NO | NO | NO |

OHDSI vocabulary version: v5.0 04-FEB-22

## End-of-life conversations: target

**Cohort Entry Events**

**People may enter the cohort when observing any of the following:**

- visit occurrences of 'GP visit'.

**Inclusion Criteria**

- Age
  - Entry events with the following event criteria: who are >= 60 years old.
- Time period
  - Entry events with the following event criteria: starting on or after January 1, 2016 and ending before January 1, 2021.
- COPD, HF, cancer
  - Entry events with any of the following criteria:
  - having at least 1 condition occurrence of 'COPD', starting between 365 days before and 1 days after cohort entry start date; allow events outside observation period.
  - having at least 1 condition occurrence of 'CHF', starting between 365 days before and 1 days after cohort entry start date; allow events outside observation period.
  - having at least 1 condition occurrence of 'Malignancy', starting between 365 days before and 1 days after cohort entry start date; allow events outside observation period.
- Limit qualifying entry events to the earliest event per person.

**Cohort Exit**

- The cohort end date will be offset from index event's start date plus 0 days.

**Cohort Eras**

- Entry events will be combined into cohort eras if they are within 0 days of each other.

**Concept Set Definitions**

- CHF

| Concept Id | Concept name | Domain | Vocabulary | Excluded | Descendants | Mapped |
| --- | --- | --- | --- | --- | --- | --- |
| 316139 | Heart failure | Condition | SNOMED | NO | YES | NO |

- COPD

| Concept Id | Concept name | Domain | Vocabulary | Excluded | Descendants | Mapped |
| --- | --- | --- | --- | --- | --- | --- |
| 255573 | Chronic obstructive lung disease | Condition | SNOMED | NO | YES | NO |

- GP visit

| Concept Id | Concept name | Domain | Vocabulary | Excluded | Descendants | Mapped |
| --- | --- | --- | --- | --- | --- | --- |
| 9202 | Outpatient Visit | Visit | Visit | NO | NO | NO |
| 581476 | Home Visit | Visit | Visit | NO | NO | NO |

- Malignancy

| Concept Id | Concept name | Domain | Vocabulary | Excluded | Descendants | Mapped |
| --- | --- | --- | --- | --- | --- | --- |
| 443392 | Malignant neoplastic disease | Condition | SNOMED | NO | YES | NO |

OHDSI vocabulary version: v5.0 04-FEB-22

## End-of-life conversations: outcome

**Cohort Entry Events**

**People may enter the cohort when observing any of the following:**

- procedure occurrence of any procedure (including 'levenseindegesprek' source concepts) for the first time in the person's history.
- procedure occurrence of 'advanced care planning' for the first time in the person's history.
- Limit cohort entry events to the earliest event per person.

**Inclusion Criteria**

- Age
  - Entry events with the following event criteria: who are >= 60 years old.
- Time period
  - Entry events with the following event criteria: starting on or after January 1, 2016.

**Cohort Exit**

The cohort end date will be offset from index event's start date plus 0 days.

**Cohort Eras**

Entry events will be combined into cohort eras if they are within 0 days of each other.

**Concept Set Definitions**

- Levenseindegesprek (End of life conversation)

| Concept Id | Concept name | Domain | Vocabulary | Excluded | Descendants | Mapped |
| --- | --- | --- | --- | --- | --- | --- |
| 2073012000 | Euthanasia request/discussion | Condition/Procedure | ICPC-1 | NO | NO | NO |

- advanced care planning

| Concept Id | Concept name | Domain | Vocabulary | Excluded | Descendants | Mapped |
| --- | --- | --- | --- | --- | --- | --- |
| 37017346 | Advance care planning | Procedure | SNOMED | NO | NO | NO |

OHDSI vocabulary version: v5.0 04-FEB-22

## Asthma exacerbation: target

**Cohort Entry Events**

**People may enter the cohort when observing any of the following:**

- condition occurrences of 'Asthma'.

**Inclusion Criteria**

- Age
  - Entry events with the following event criteria: who are >= 18 years old.
- Time period
  - Entry events with the following event criteria: starting on or after January 1, 2015 and ending before January 1, 2020.
- COPD
  - Entry events having no condition occurrences of 'COPD', starting anytime up to 365 days after cohort entry start date; allow events outside observation period.
- Treatment
  - Entry events having at least 1 drug exposure of 'R03 drugs', starting between 30 days before and 30 days after cohort entry start date.
- Limit qualifying entry events to the earliest event per person.

**Cohort Exit**

- The cohort end date will be offset from index event's start date plus 0 days.

**Cohort Eras**

- Entry events will be combined into cohort eras if they are within 0 days of each other.

**Concept Set Definitions**

- Asthma

| **Concept Id** | **Concept name** | **Domain** | **Vocabulary** | **Excluded** | **Descendants** | **Mapped** |
| --- | --- | --- | --- | --- | --- | --- |
| 317009 | Asthma | Condition | SNOMED | NO | YES | NO |

- COPD

| **Concept Id** | **Concept name** | **Domain** | **Vocabulary** | **Excluded** | **Descendants** | **Mapped** |
| --- | --- | --- | --- | --- | --- | --- |
| 255573 | Chronic obstructive lung disease | Condition | SNOMED | NO | YES | NO |

- R03 drugs

| **Concept Id** | **Concept name** | **Domain** | **Vocabulary** | **Excluded** | **Descendants** | **Mapped** |
| --- | --- | --- | --- | --- | --- | --- |
| 21603248 | DRUGS FOR OBSTRUCTIVE AIRWAY DISEASES | Drug | ATC | NO | YES | NO |

OHDSI vocabulary version: v5.0 04-FEB-22

## Asthma exacerbation: outcome

**Cohort Entry Events**

**People may enter the cohort when observing any of the following:**

- drug eras of '[AM] Systemic glucocorticoids 8-3', with era length between 3 and 30 days.

**Inclusion Criteria**

- Age
  - Entry events with the following event criteria: who are >= 18 years old.
- Time period
  - Entry events with the following event criteria: starting on or after January 1, 2016.

**Cohort Exit**

- The cohort end date will be offset from index event's start date plus 0 days.

**Cohort Eras**

- Entry events will be combined into cohort eras if they are within 0 days of each other.

**Concept Set Definitions**

- Systemic glucocorticoids 8-3
  - All medication with listed ingredient and dose from:

| **ATC code** | **Ingredient** | **Concept Id** | **Included dose forms** |
| --- | --- | --- | --- |
| H02AB01 | betamethasone | 920458 | Injectable, oral |
| H02AB02 | dexamethasone | 1518254 | Injectable, oral |
| H02AB03 | fluocortolone | 19055344 | Injectable, oral |
| H02AB04 | methylprednisolone | 1506270 | Injectable, oral |
| H02AB05 | paramethasone | 19027186 | Injectable, oral |
| H02AB06 | prednisolone | 1550557 | Injectable, oral |
| H02AB07 | prednisone | 1551099 | Injectable, oral |
| H02AB08 | triamcinolone | 903963 | Injectable, oral |
| H02AB09 | hydrocortisone | 975125 | Injectable, oral |
| H02AB10 | cortisone | 1507705 | Injectable, oral |
| H02AB11 | prednylidene | 19011127 | Injectable, oral |
| H02AB12 | rimexolone | 977421 | Injectable, oral |
| H02AB13 | deflazacort | 19086888 | Injectable, oral |
| H02AB14 | cloprednol | 19050907 | Injectable, oral |
| H02AB15 | meprednisone | 19009116 | Injectable, oral |
| H02AB17 | cortivazol | 19061907 | Injectable, oral |
| H02AB90 | flumetasone | 19055156 | Injectable, oral |

OHDSI vocabulary version: v5.0 04-FEB-22

## Mortality in COPD: outcome

**Cohort Entry Events**

**People may enter the cohort when observing any of the following:**

- death of any form.
- observations of 'Death'.
- Limit cohort entry events to the earliest event per person.

**Inclusion Criteria**

- Age
  - Entry events with the following event criteria: who are >= 40 years old.
- Time period
  - Entry events with the following event criteria: starting on or after January 1, 2015.

**Cohort Exit**

- The cohort end date will be offset from index event's start date plus 0 days.

**Cohort Eras**

- Entry events will be combined into cohort eras if they are within 0 days of each other.

**Concept Set Definitions**

- Death

| **Concept Id** | **Concept name** | **Domain** | **Vocabulary** | **Excluded** | **Descendants** | **Mapped** |
| --- | --- | --- | --- | --- | --- | --- |
| 4306655 | Death | Observation | SNOMED | NO | YES | NO |

OHDSI vocabulary version: v5.0 04-FEB-22

## Mortality in COPD: target

**Cohort Entry Events**

**People may enter the cohort when observing any of the following:**

- condition occurrence of 'COPD' for the first time in the person's history.
- Limit cohort entry events to the earliest event per person.

**Inclusion Criteria**

- Age
  - Entry events with the following event criteria: who are >= 40 years old.
- Time period
  - Entry events with the following event criteria: starting on or after January 1, 2015 and ending before January 1, 2020.

**Cohort Exit**

- The cohort end date will be offset from index event's start date plus 0 days.

**Cohort Eras**

- Entry events will be combined into cohort eras if they are within 0 days of each other.

**Concept Set Definitions**

- COPD

| **Concept Id** | **Concept name** | **Domain** | **Vocabulary** | **Excluded** | **Descendants** | **Mapped** |
| --- | --- | --- | --- | --- | --- | --- |
| 255573 | Chronic obstructive lung disease | Condition | SNOMED | NO | YES | NO |

OHDSI vocabulary version: v5.0 04-FEB-22
