# Supplementary material for: The added value of text from Dutch general practitioner notes in predictive modeling
Source: J Am Med Inform Assoc. 2023 Aug 16;30(12):1973–84. doi: 10.1093/jamia/ocad160 (PMC10654855; doi:10.1093/jamia/ocad160)
Supplement: ocad160_Supplementary_Data [file ocad160_supplementary_data.zip › ocad160_Supplementary_Data/PlpUnstructuredData_supplement_FigureAndTables.docx]

# Supplementary material

## Tables

**Table S1**. Overview of Dutch vocabularies used as reference for the clinical concept extraction.

| **Abbreviation** | **Name** | **Source** |
| --- | --- | --- |
| SNOMED CT | SNOMED Clinical Terms and patient friendly terms | Dutch National IT Institute for Healthcare (NICTIZ) |
| MeSH | Medical Subject Headings | Unified Medical Language System (UMLS) |
| ICD10 | International Classification of Diseases 10^th^ revision | Unified Medical Language System (UMLS) |
| ICPC-1 | International Classification of Primary Care | Unified Medical Language System (UMLS) |
| MedDRA | Medical Dictionary for Regulatory Activities | Unified Medical Language System (UMLS) |
| LOINC | Logical Observation Identifiers Names and Codes | Unified Medical Language System (UMLS) |

**Table S2**. Overview of the three machine learning models, their hyperparameters, and hyperparameter values.

| **Model** | **Hyperparameter** | **Range** |
| --- | --- | --- |
| lasso regularized logistic regression (LR) | Variance | 0.01 – 20 |
| Extreme gradient boosting (XGB) | Number of trees | 100, 300 |
|  | Maximum depth | 4, 6, 10 |
|  | Learning rate | 0.01, 0.1 |
| Random forest (RF) | Number of trees | 100, 500 |
|  | Maximum depth | 5, 10, 20 |
|  | Minimum sample split | 2, 5 |
|  | Minimum samples leaf | 1, 10 |

**Table S3**. Overview of the different methods used for training the prediction models.

| **Method** | **Options** |
| --- | --- |
| Structured data features | Demographics (*D*) |
|  | Condition, drug, measurement, and procedure events (*E*) |
| Text representations | Binary bag-of-words (*Tterm*)  TFIDF bag-of-words (*Ttfidf*) |
|  | Extracted clinical concepts (*Tcon*) |
| Machine learning algorithms | L1 Regularized logistic regression (*LR*) |
|  | Extreme gradient boosting (*XGB*) |
|  | Random forest (*RF*) |
| Feature set combinations | **S:** Demographics (*D*) + clinical events (*E*) |
|  | **T:** Demographics (*D*) + a text representation (*Tterm, Ttfidf,* or *Tcon*) |
|  | **S+T**: Demographics (*D*) + clinical events (*E*) + a text representation (*Tterm, Ttfidf,* or *Tcon*) |

## Figures


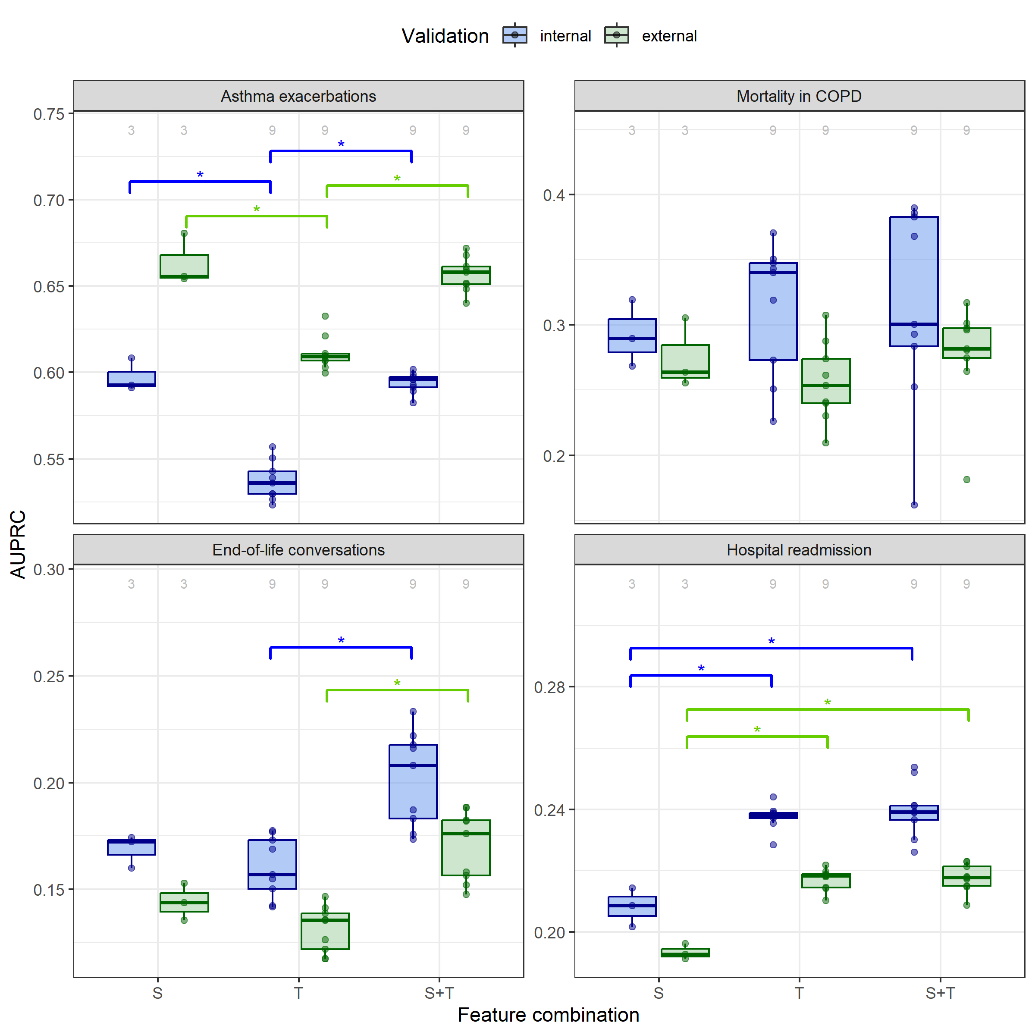


**Figure S1**. Distribution of the AUPRC values of the internal validation (blue) and external validation (green) for models trained using the different feature combinations: structured feature set (S), text feature sets (T), and combined feature sets (S+T), per prediction task. The number of models in each boxplot is indicated above the boxplot. The significant Bonferroni adjusted Wilcoxon test results between the feature combinations are shown above the boxplots, where ‘*’ indicates a p-value<0.05. The points represent the underlying data.


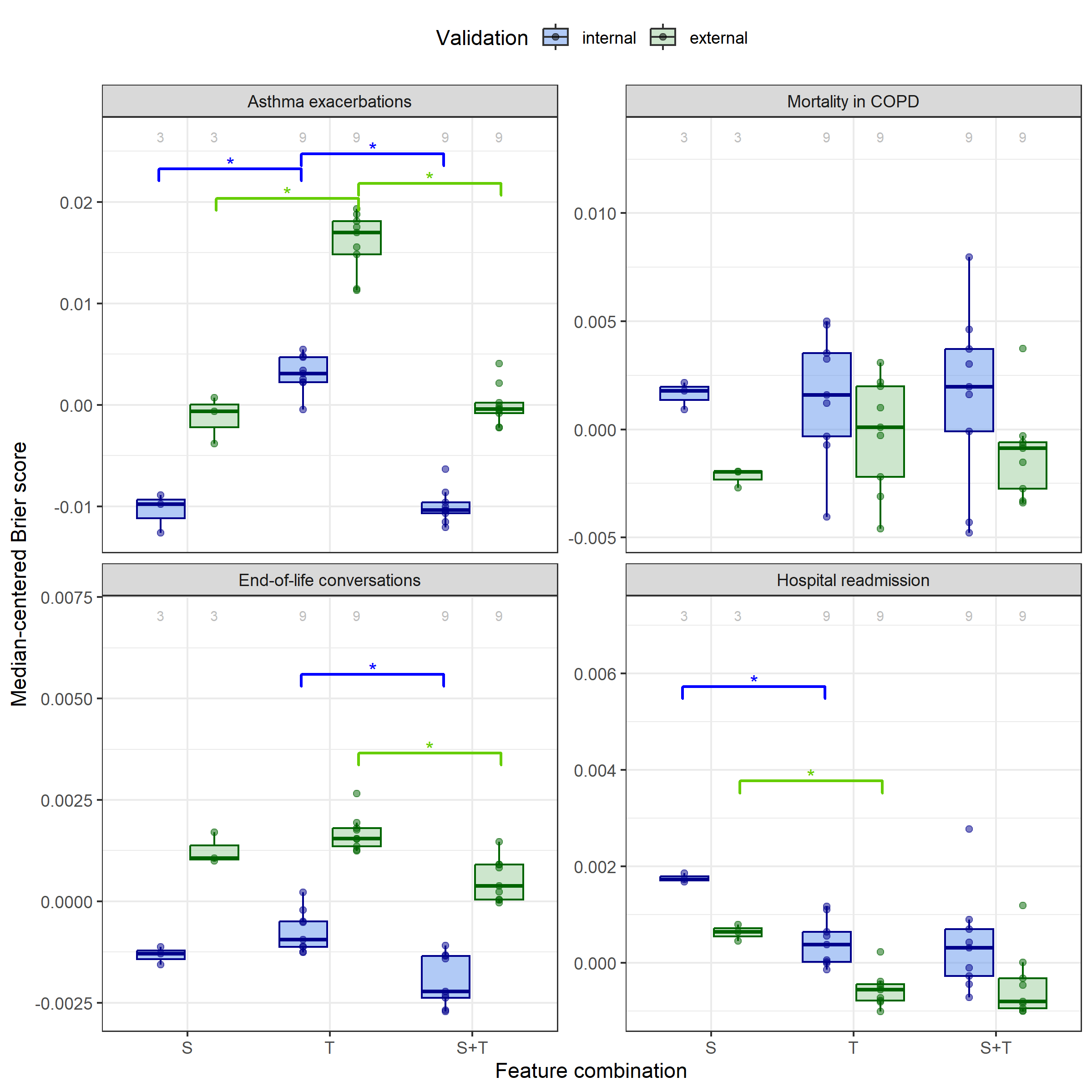


**Figure S2**. Distribution of the Brier scores of the internal validation (blue) and external validation (green) for models trained using the different feature combinations: structured feature set (S), text feature sets (T), and combined feature sets (S+T), per prediction task. The number of models in each boxplot is indicated above the boxplot. The significant Bonferroni adjusted Wilcoxon test results between the feature combinations are shown above the boxplots, where ‘*’ indicates a p-value<0.05. The points represent the underlying data.


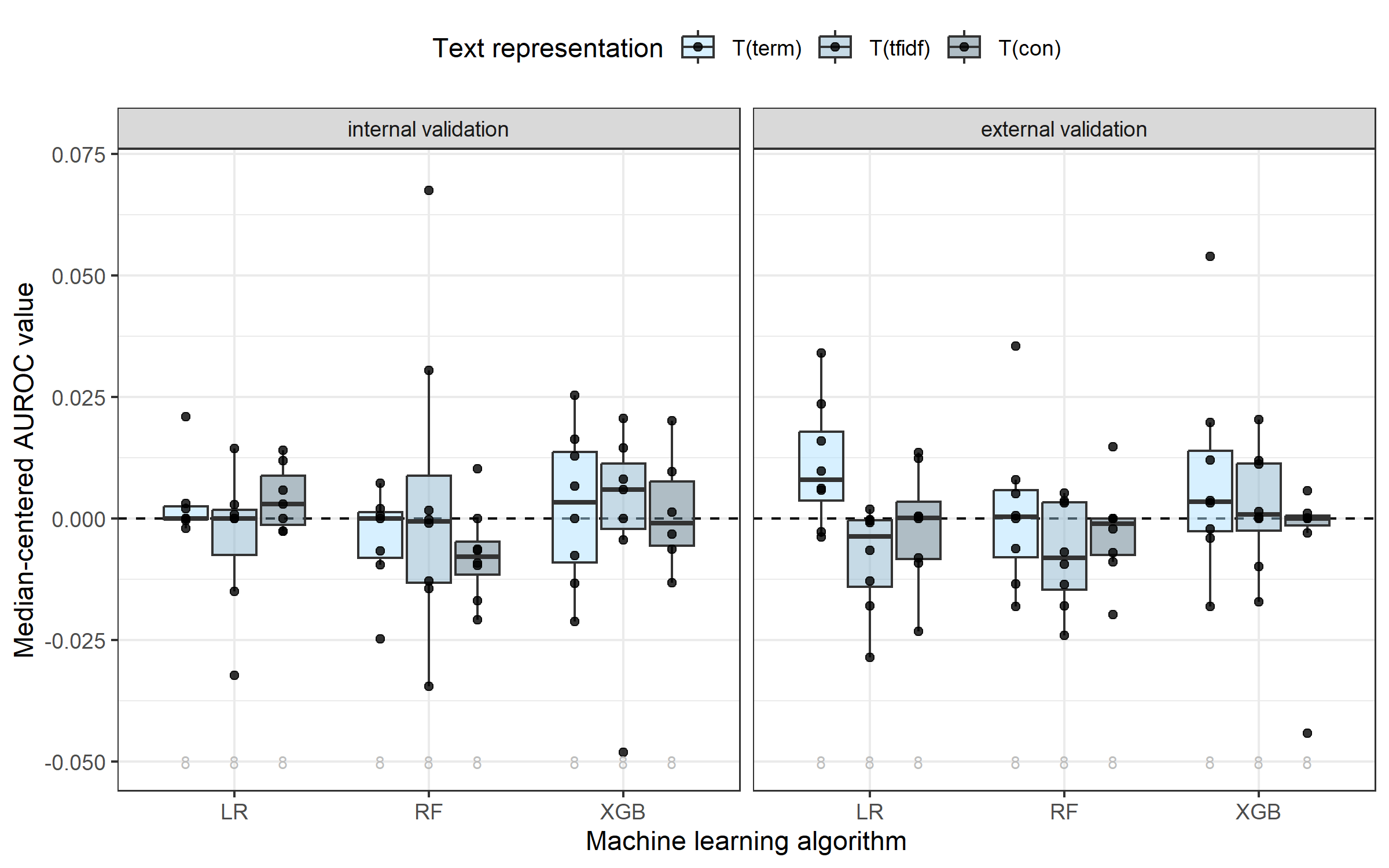


**Figure S3**. Distributions of the median-centered AUPRC value for the different text representations and machine learning algorithms. The plots on the left-hand side present the internal validation and the plots on the right-hand side present the external validation. The number of models is noted below each boxplot. No significant Bonferroni adjusted Wilcoxon test results between the text representation and machine learning algorithm combinations were found.
